# Unravelling the Proteomics of HLA-B*57:01^+^ Antigen Presenting Cells during Abacavir Medication

**DOI:** 10.3390/jpm12010040

**Published:** 2022-01-04

**Authors:** Funmilola Josephine Haukamp, Eline Gall, Gia-Gia Toni Hò, Wiebke Hiemisch, Florian Stieglitz, Joachim Kuhn, Rainer Blasczyk, Andreas Pich, Christina Bade-Döding

**Affiliations:** 1 Institute for Transfusion Medicine and Transplantat Engineering, Hannover Medical School, Carl-Neuberg-Str. 1, 30625 Hannover, Germany; Gall.Eline@mh-hannover.de (E.G.); Ho.Gia-Gia@mh-hannover.de (G.-G.T.H.); Hiemisch.Wiebke@mh-hannover.de (W.H.); Blasczyk.Rainer@mh-hannover.de (R.B.); Bade-Doeding.Christina@mh-hannover.de (C.B.-D.); 2 Institute of Toxicology, Hannover Medical School, Carl-Neuberg-Str. 1, 30625 Hannover, Germany; Stieglitz.Florian@mh-hannover.de (F.S.); Pich.Andreas@mh-hannover.de (A.P.); 3 Core Facility Proteomics, Hannover Medical School, Carl-Neuberg-Str. 1, 30625 Hannover, Germany; 4 Institute for Laboratory and Transfusion Medicine, Heart and Diabetes Center North Rhine-Westphalia, Ruhr University Bochum, Georgstr. 11, 44801 Bochum, Germany; JKuhn@hdz-nrw.de

**Keywords:** adverse drug reaction, HLA-B*57:01, abacavir, proteome, hypersensitivity

## Abstract

Type B adverse drug reactions (ADRs) are unpredictable based on the drug’s pharmacology and represent a key challenge in pharmacovigilance. For human leukocyte antigen (HLA)-mediated type B ADRs, it is assumed that the protein/small-molecule interaction alters the biophysical and mechanistic properties of the antigen presenting cells. Sophisticated methods enabled the molecular appreciation of HLA-mediated ADRs; in several instances, the drug molecule occupies part of the HLA peptide binding groove and modifies the recruited peptide repertoire thereby causing a strong T-cell-mediated immune response that is resolved upon withdrawal of medication. The severe ADR in HLA-B*57:01^+^ patients treated with the antiretroviral drug abacavir (ABC) in anti-HIV therapy is an example of HLA-drug-T cell cooperation. However, the long-term damages of the HLA-B*57:01-expressing immune cells following ABC treatment remain unexplained. Utilizing full proteome sequencing following ABC treatment of HLA-B*57:01^+^ cells, we demonstrate stringent proteomic alteration of the HLA/drug presenting cells. The proteomic content indisputably reflects the cellular condition; this knowledge directs towards individual pharmacovigilance for the development of personalized and safe medication.

## 1. Introduction

Adverse drug reactions (ADRs) are harmful and unintended reactions triggered by a drug used in the prevention, diagnosis or therapy of diseases at suitable pharmacological dosage [1]. They occur despite proper application of the respective drug and are classified as type A and type B reactions based on their pharmacological predictability [2]. Type A ADRs arise dose-dependently and are triggered by pharmacodynamic reactions and influenced by pharmacokinetics [3,4], hence these augmented reactions are rarely life-threatening. In contrast, type B reactions are not predictable based on the drug’s pharmacology and seem to be idiosyncratic. Both the innate and the adaptive immune system can be involved in the development of an idiosyncratic ADR; they occur especially in patients with a certain predisposition and result in high mortalities [5,6]. Manifestations comprise cutaneous symptoms ranging from milder exanthema to life-threatening Stevens–Johnson Syndrome (SJS) or toxic epidermal necrolysis (TEN), yet liver, kidney and blood cells might be affected as well [7].

Type B reactions are further divided into (1) immediate onset antibody-mediated reactions that cause rapid and serious allergic reaction like anaphylaxis and (2) delayed hypersensitivity reactions (DHRs) characterized by an onset of symptoms within hours or weeks post drug administration [8]. DHRs are often T-cell-mediated and triggered by drug-induced immune stimulation [9,10]. In recent years, associations between type B ADRs and distinct alleles of the highly polymorphic human leukocyte antigen (HLA) system have been discovered [11,12,13,14]. These cell surface molecules are involved in immune responses through the presentation of endogenously processed self- or pathogen-derived peptides to autologous T cells. In the case of HLA class I molecules that are expressed on almost all nucleated cells [15], the self-peptidome is presented to CD8^+^ T cells that recognize the trimeric complex of HLA class I heavy chain, light chain and peptide with their respective T cell receptors (TCRs) [16]. The simultaneous co-recognition of a TCR for self HLA class I molecules and peptides of self or foreign origin is unique in receptor-ligand interactions [17]. Based on the origin of the presented peptide, the immune system appreciates the individual health condition and elicits immune responses. Polymorphisms within distinct HLA allotypes result in differential structural and electrochemical features of the peptide binding region (PBR) and therefore determine the presented immunopeptidome [18].

Synthetic substances used as medical products are usually too small (<1 kDa) to directly induce an immune response. However, three hypotheses have been proposed to explain how small molecules can activate the immune system in an HLA-dependent manner: by (1) operating as a hapten or prohapten that covalently reacts with endogenous proteins or peptides resulting in a haptenated, de novo product with immunogenic features (hapten/prohapten model); (2) binding to immune receptor proteins (HLA and/or TCR) themselves in a direct, reversible and non-covalent manner (p-i model); or (3) occupying the PBR of distinct HLA alleles during HLA assembly resulting in new peptide binding motifs and the presentation of novel self-peptides as neo-antigens (altered repertoire model) [2,19]. The small molecule/HLA interaction might be drug-specific and HLA type-specific, the unique occurrence of one theoretical model or the exclusion of one theoretical model cannot be specified.

Abacavir (ABC) is a drug used as part of a highly active antiretroviral therapy (HAART) for the treatment of HIV-1^+^ patients. Approximately 5% of HIV-1^+^ patients develop DHRs post ABC treatment. This Abacavir Hypersensitivity Syndrome (AHS) is characterized by multiorgan symptoms including fever, gastrointestinal complaints, and skin rash usually appearing during the first 42 days of treatment with a median time to onset of 11 days [20]. Symptoms usually resolve 24 h after discontinuation of ABC treatment, however, if patients are re-challenged with ABC, AHS would rapidly recur with potential life-threatening consequences [21].

In the early 2000s, several clinical studies found a strong predictive association between AHS and the occurrence of the genotype HLA-B*57:01 [22,23,24]. Thus, HLA typing and screening for HLA-B*57:01 is recommended by the Food and Drug Administration (FDA) drug label of ABC since 2008 and the presence of HLA-B*57:01 is a contraindication for treatment of HIV-1^+^ patients with ABC [25].

The mechanism that enables ABC to activate the immune system and elicit immune responses corresponds to the altered repertoire model, this could be demonstrated unambiguously by structural evidence. ABC binds specifically within the PBR of HLA-B*57:01 and alters the biochemical features of the HLA F pocket. As a result, an altered peptide binding motif allows novel self-peptides to bind to HLA-B*57:01 and to be presented to autologous CD8^+^ T cells [26,27,28]. Due to missing T cell tolerance to these unknown self-peptides, autologous CD8^+^ T cells start eliciting polyclonal T cell responses against the neo-antigen [29].

Before admission, a medical product undergoes several approval filters. As these clinical trials include a selected and limited number of patients, it becomes obvious that the occurrence of adverse events are often overlooked or misinterpreted. Yet, there are numerous medical products that are strongly affected by the individual genetics of a patient. Knowledge about the full protein spectrum that is influenced by a drug contributes to the identification of potential risk factors and hence, is extremely important for drug safety. Full proteome analysis is a sophisticated methodology to elucidate the impact of drug treatment on cells [30].

ABC is an excellent candidate to exploit the mechanism and find possible prognostic markers responsible for HLA-associated ADRs due to the extraordinary specificity of AHS and HLA-B*57:01 [31]. This study aims to conduct an in vitro investigation into the cellular response to ABC using proteomic strategies to increase the knowledge of molecular processes involved in the development of AHS. Comprehensive understanding will provide significant fundamental insight in the area of immunology that will allow the prediction of a patient’s risk for HLA-mediated ADRs and contribute to well-advanced personalized and safe treatments.

## 2. Materials and Methods

### 2.1. Maintenance of Cell Lines

All cell lines were cultured at 37 °C and 5% CO_2_. The human B lymphoblastoid cell line *LCL721.221* (LGC Promochem^®^, Wesel, Germany; HLA class I^-^/TPN^+^) was cultured in RPMI 1640 medium (Lonza, Basel, Switzerland) supplemented with 10% heat-inactivated fetal calf serum (FCS, Lonza), 2 mM L-glutamine (c. c. pro, Oberdorla, Germany), 100 U/mL penicillin and 100 µg/mL streptomycin (c. c. pro).

The human embryonal kidney cell line *HEK239T* (cell source ATCC, Manassas, VA, USA) was grown in Dulbecco’s Modified Eagle Medium (DMEM, Lonza) supplemented with 10% heat-inactivated FCS, 2 mM L-glutamine, 100 U/mL penicillin, 100 µg/mL streptomycin and 1 mg/mL Geneticin^®^ (Life Technologies, Carlsbad, CA, USA).

### 2.2. Cloning of Constructs Encoding for Soluble HLA-B*57:01

Constructs encoding for full length HLA-B*57:01 (mHLA-B*57:01, Exon 1–7) were generated from cDNA of an HLA-B*57:01^+^ donor via PCR as previously described [32]. The cDNA encoding for soluble HLA-B*57:01 (sHLA-B*57:01, Exon 1–4) was generated by side-directed mutagenesis and cloned into the lentiviral vector pRRL.PPT.SFFV.mcs.pre and checked through sequencing.

### 2.3. Stable Transduction of LCL721.221 Cells with Lentivirus Encoding for sHLA-B*57:01

*HEK293T* cells were transfected with the sHLA-B*57:01 encoding transfer vector and the packaging and envelope vectors psPAX and pmD2G. Produced virus was used to stably transduce *LCL721.221* cells as previously described [33,34]. The expression of sHLA-B*57:01 molecules was confirmed by ELISA as described by Celik et al. [35].

### 2.4. Mass Spectrometric Detection of ABC in Solution

For ABC tracking in cell lysates, 5 × 10^5^ *LCL721.221* and LCL721.221/sHLA-B*57:01 cells were incubated with or without 50 µg/mL ABC for 48 h. Cells were lysed in 250 µL RIPA buffer as described by Ho et al. [36]. For sample preparation, 50 μL of sample, standard or quality control was added to 200 μL 0.1 M ZnSO_4_. For precipitation, 500 μL of internal standard (IS) precipitation solution was added to the mixture and mixed for 10 s, followed by a centrifugation step at 13,000× *g* for 10 min. The clear supernatant was transferred into MS-vials and 1.0 μL was injected into the UPLC-MS/MS system.

ABC was separated on a 2.1 × 50-mm reverse phase column (Waters, Acquity UPLC BEH Phenyl, 1.7 μm) maintained at 50 °C using an ultra-performance liquid chromatography system directly coupled to a Waters TQ electrospray ionization-tandem mass spectrometry (TQD). The sample was applied at a flow rate of 0.5 mL/min using the following gradient program: isocratic flow of 75%/25% water/methanol containing 0.1% formic acid and 2 mmol/L ammonium acetate was performed for 60 s. After that a linear gradient over 1.3 min of 5%/95% water/methanol containing 0.1% formic acid and 2 mmol/L ammonium acetate followed. After the isocratic elution of 95% methanol for 0.5 min, the mobile phase was reversed to the initial state.

The TQD was operated in electrospray positive ionization mode. The system controls of the devices and data acquisition were performed using *MassLynx NT 4.1* software. Data processing was performed by the *MassLynx QuanLynx* program. Nitrogen was used as the nebulizing gas and argon as the collision gas. Instrument settings were as follows: capillary voltage, 0.423 kV; source temperature, 110 °C; desolvation temperature, 480 °C; collision gas pressure, 2.6 × 10^−3^. Samples analysis was performed in the multiple reaction monitoring mode (MRM) of the instrument. Sample cone voltage, collision energy, dwell time, and mass transitions for all compounds are listed in Table 1.

### 2.5. Mass Spectrometric Analysis of ABC-Induced Modifications of the LCL721.221 Proteome

For proteome analysis, 1 × 10^6^ *LCL721.221* and LCL721.221/sHLA-B*57:01 cells were treated with 50 µg/mL ABC for 48 h. Drug treatment was repeated after 24 h. Cells were harvested and lysed in RIPA buffer as previously described. By using the Bicinchoninic Acid Assay (BCA) Protein Quantitation Kit (Interchim, San Diego, CA, USA) protein concentration was determined.

Sample preparation and MS analysis was performed as previously described by Simper et al. [37]. Proteins were digested in solution with Lys-C for 4 h at 37 °C and afterwards with trypsin overnight at 37 °C.

## 3. Results

### 3.1. Development of a Mass Spectrometric Method for the Detection of ABC in Solution

**Figure 1:**
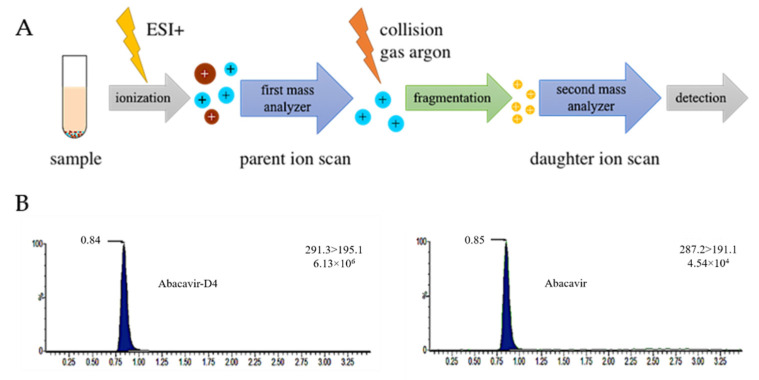
MS/MS method for the detection of ABC. (**A**) Principle of a tandem mass spectrometer. (**B**) MRM chromatograms of the first mass transition for analyte quantification of ABC-D_4_ and ABC.

For the detection of ABC in solution, a tandem mass spectrometry (MS/MS) method using multiple reaction monitoring (MRM) mode for sample analysis was established. The principle of the MS/MS method we used is the following: the sample that was previously separated by ultra-performance liquid chromatography (UPLC) is ionized by positive electrospray ionization (ESI+). The ionized precursor/parent ion masses are scanned in the first mass analyzer (parent ion scan). Only molecule–ions with a uniquely defined mass find their way to the collision cell where the collision gas argon leads to a fragmentation of these molecule–ions. Fragmented daughter ions are analyzed in the second mass analyzer (daughter ion scan) (Figure 1A). Though every molecule features a characterized fragmentation pattern, this method offers the possibility to identify a specific analyte. To maximize the peak area corresponding to the molecular mass of the analyte ABC and its daughter ions and to achieve the desired response for ABC, MS settings (capillary and cone voltage and desolvation temperature) were optimized. The adjusted MS settings were used to establish a MS/MS method using MRM (Table 1). For ABC and deuterated ABC (ABC-D_4_, used as internal standard), the two most sensitive mass transitions were utilized for determination. The first mass transition allowed for quantification of the analyte. The second mass transition leads to an increase in specificity of the method. Figure 1B illustrates the MRM chromatogram of the most sensitive daughter ion for quantification of ABC and ABC-D_4_.

### 3.2. Recombinant B Cells Absorb ABC

**Figure 2:**
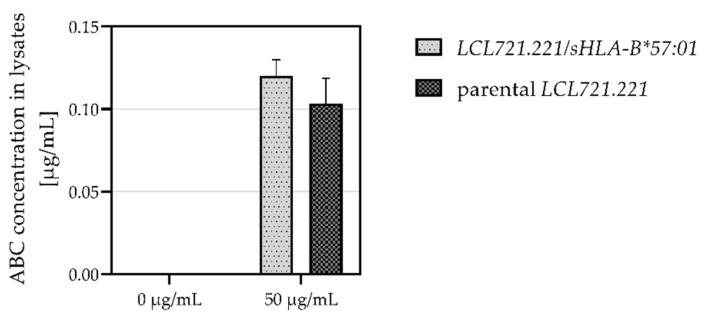
ABC concentration in parental *LCL721.221* and LCL721.221/sHLA-B*57:01 cell lysates. Cells were incubated without or with 50 µg/mL ABC for 48 h and ABC concentrations were measured in cell lysates of three technically independent replicates (*n* = 3) by UPLC-MS/MS.

To assure ABC uptake in recombinant B cells, parental *LCL721.221* and *LCL721.221* cells expressing soluble HLA-B*57:01 (LCL721.221/sHLA-B*57:01) were treated without or with 50 µg/mL ABC and cultured for 48 h. Afterwards, ABC concentration in cell lysates was measured. In LCL721.221/sHLA-B*57:01 cells 0.12 µg/mL ABC and in parental *LCL721.221* cells 0.10 µg/mL ABC was detected (Figure 2). Additionally, cell viability of *LCL721.221* cells treated with ABC was verified (Figure S1). The absorption of ABC in recombinant B cells was verified and ABC absorption capacity was similar in parental *LCL721.221* and LCL721.221/sHLA-B*57:01 cells.

### 3.3. Impact of ABC Treatment on Protein Expression of Recombinant B Cells

The cellular response of LCL721.221/sHLA-B*57:01 cells and parental *LCL721.221* cells to ABC was investigated by full proteome analysis. The respective recombinant B cell lines were cultivated either without or with ABC for 48 h. After 24 h ABC treatment was repeated. Label free quantification enabled the determination of relative protein abundance and relative protein amounts were calculated by *MaxQuant* software. Each cell line was normalized by subtracting the median value from each sample.

**Figure 3:**
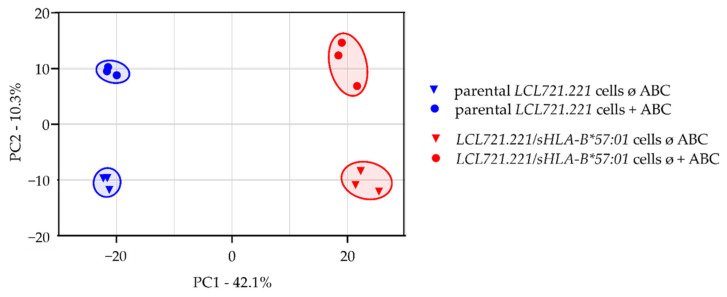
Principal component analysis (PCA) of proteins that were significantly altered (*p* < 0.05) in parental *LCL721.221* cells (blue) and LCL721.221/sHLA-B*57:01 cells (red) without ABC treatment (circle) and following ABC treatment (triangle). Cells were incubated without (ø) or with (+) ABC for 48 h in three technically independent replicates (*n* = 3).

For visualization of significant differences between both cell lines as well as no treatment and ABC treatment, a principal component analysis (PCA) was performed. Significant differences between all conditions are detectable (Figure 3).

Due to differences of the proteomic content in parental *LCL721.221* and LCL721.221/sHLA-B*57:01 cells that might have arisen through lentiviral transduction of the cells, a further normalization step was applied by subtracting the proteome content of untreated cells from ABC-treated cells. Thereby proteomic differences due to previous lentiviral transduction of LCL721.221/sHLA-B*57:01 cells were eliminated and comparison of both cell lines solely among ABC treatment was enabled.

**Figure 4:**
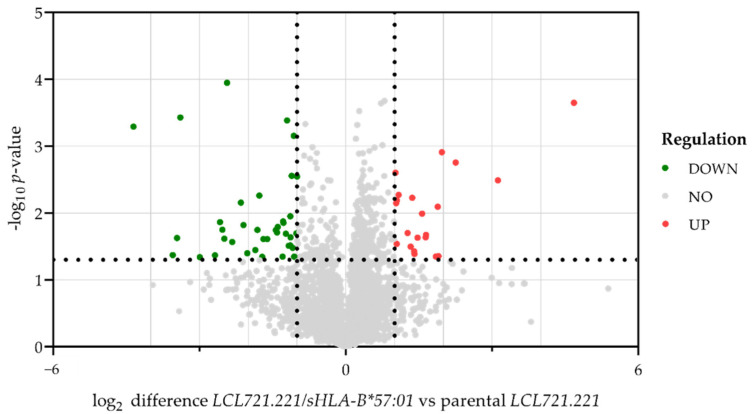
Differences in protein abundance in LCL721.221/sHLA-B*57:01 cells compared to parental *LCL721.221* cells following ABC treatment. The volcano plot illustrates significantly differentially abundant proteins after ABC treatment of three technically independent replicates (*n* = 3). The log_2_-fold change of LCL721.221/sHLA-B*57:01 cells compared to parental *LCL721.221* cells is plotted against the –log_10_ *p*-value. Proteins were regarded as regulated in LCL721.221/sHLA-B*57:01 cells from factor ± 1.0 and *p* < 0.05. Downregulated proteins are labelled in green; unregulated proteins are colored in grey and upregulated proteins are given in red.

LC-MS-based proteomic analysis of LCL721.221/sHLA-B*57:01 cells compared to parental *LCL721.221* cells following ABC treatment revealed 5620 identified protein groups; 3880 of them could be quantified. Exclusively significant regulated proteins (*p*-value < 0.05) and proteins that were at least altered by a factor of log_2_ ± 1.0 in LCL721.221/sHLA-B*57:01 cells compared to parental *LCL721.221* cells were regarded as regulated. In total, 60 quantified proteins were significantly regulated; 21 of them were significantly upregulated in LCL721.221/sHLA-B*57:01 cells compared to parental *LCL721.221* cells (Figure 4, depicted in red).

The 10 strongest upregulated proteins following ABC treatment of LCL721.221/sHLA-B*57:01 cells compared to parental *LCL721.221* cells are depicted in Table 2. The strongest upregulated protein is the histone variant H3.3 (H3F3A) that substitutes conventional H3.3 in a wide range of transcriptionally active chromatin [38]. Furthermore, the nucleoside diphosphate kinase A (NME1) that is involved in cell proliferation, differentiation and development as well as in signal transduction and gene expression is also significantly upregulated. The interferon-induced helicase C domain-containing protein 1 (IFIH1) belongs to the pattern recognition receptors (PRRs) and recognizes viral infection resulting in the activation of antiviral responses shows a 4.75-fold upregulation in ABC-treated LCL721.221/sHLA-B*57:01 cells compared to parental cells.

**Figure 5:**
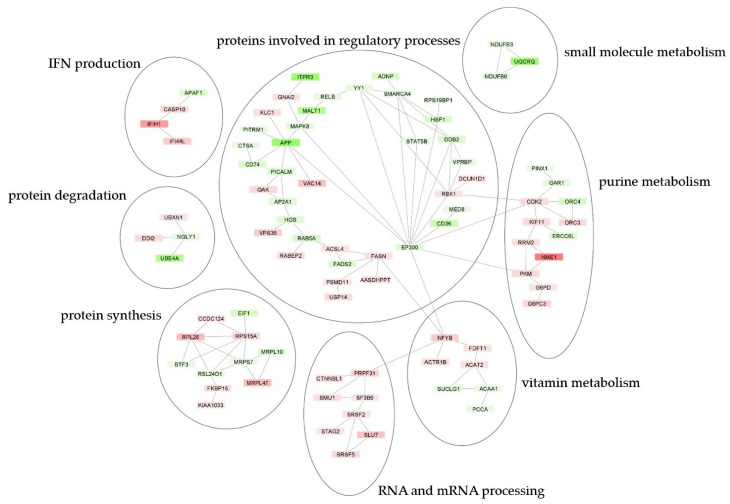
Protein–protein interaction network of significantly up- and downregulated proteins following ABC treatment in LCL721.221/sHLA-B*57:01 cells compared to parental *LCL721.221* cells and cellular processes they are involved in (based on GO/KEGG). Network was constructed by STRING Database (Version 11.5) and visualized using Cytoscape (Version 3.8.2). Significant regulated proteins (*p* < 0.05) at least altered by a factor of log_2_ ± 0.5 were considered. Upregulated proteins are illustrated in red; downregulated proteins are depicted in green. Color intensity reflects the log_2_-fold difference between LCL721.221/sHLA-B*57:01 cells compared to parental *LCL721.221* cells.

A protein–protein interaction (PPI) network created via STRING Database demonstrated the involvement of significantly regulated proteins in LCL721.221/sHLA-B*57:01 cells compared to parental cells following ABC treatment in various cellular processes (Figure 5). Upregulated proteins are particularly involved in interferon (IFN) production, RNA and mRNA processing and purine metabolism.

We utilized the *Ingenuity Pathway Analysis* (*IPA*) software to identify upstream regulators of the identified significantly up- and downregulated proteins (Table 3). The macrophage colony-stimulating factor 1 (CSF1), the histone acetyltransferase KAT2A and the interferon regulatory factor 3 (IRF3) were identified as activated in LCL721.221/sHLA-B*57:01 cells compared to parental cells following ABC treatment. CSF1 is a cytokine that has an essential function in innate immunity and inflammatory processes by promoting the release of proinflammatory chemokines upon binding to its cognate receptor. KAT2A has been described to positively regulate the activation of T cells by acetylation of histone H3 resulting in Interleukin-2 (IL-2) expression. As a transcriptional regulator of type I IFN-dependent immune responses, IRF3 has an important role in innate immune responses against viruses. In contrast, *IPA* revealed the signal transducer and activator of transcription 6 (STAT6) and the DNA binding protein Ikaros (IKZF1) to be inhibited in LCL721.221/sHLA-B*57:01 cells compared to parental *LCL721.221* cells following ABC treatment. STAT6 induces transcription activation, transduces signals and is especially involved in IL-3- and IL-4-mediated signaling. IKZF1 is involved in the regulation of lymphocyte differentiation and proliferation. Additionally, IKZF1 plays an important role in maintenance of self-tolerance.

## 4. Discussion

Pharmacovigilance pre- and post-approval of a drug is fundamental for safe medication. Currently, patient-specific personalized medication is a significant topic, since over time more and more ADRs became apparent. HLA-mediated ADRs are the most prevalent problem according to the highly polymorphic HLA genetics.

Due to the extraordinary specificity of AHS and the occurrence of the HLA-B*57:01 genotype, exploiting the mechanism of ABC-induced ADR would guide towards a deeper comprehension of HLA-mediated ADRs. The low PPV for many HLA-associated ADRs indicate that not solely the occurrence of the respective HLA risk allele, but also the involvement of other patient-specific factors might contribute to the development of type B ADRs [39].

The field of proteomics has become more and more important in assessing ADRs. The proteomic repertoire of a cell reflects a real-time view on the individual’s health status. Drugs usually interfere with the structural integrity or cellular function of its primary target, but can also interact with off-targets and effect cellular pathways that might result in phenotypic effects in the treated patient [40]. Thus, the analysis of proteomic changes induced by treatment with a certain drug contributes to earlier detection of ADRs and a decrease of health hazards in patients, and consequently improves drug safety.

In this study, we compared the regulatory impact of ABC on LCL721.221/sHLA-B*57:01 and parental *LCL721.221* target cells with particular attention to the influence of small molecule/HLA complexes on proteomic development [30]. To ensure the integrity of recombinant LCL721.221/HLA-B*57:01 target cells treated with ABC, a cytotoxicity assay according to Jedema et al. [41] was established that allowed for the detection of ABC-specific cytotoxicity of CD8^+^ cells from HLA-B*57:01 carriers towards LCL721.221/HLA-B*57:01 target cells treated with ABC (Figure S2).

Full proteome analysis revealed significant differences in the cellular response of the respective proteomes following ABC treatment. In 2012, Ostrov et al. analyzed peptide sequences eluted from untreated and ABC-treated LCL721.221/sHLA-B*57:01 cells [28]. Consistent with the peptides (KSRPEDQRSSF and RTIKKQRKY) that have been eluted solely following ABC treatment derived from Hermansky-Pudlak syndrome 5 protein (HPS5) and the mitochondrial transcription factor A (TFAM), we found HPS5 and TFAM 3-fold upregulated and thus, significantly more abundant following ABC treatment in LCL721.221/sHLA-B*57:01 cells when compared to parental *LCL721.221* cells. The significant differences in protein upregulation in response to ABC treatment between LCL721.221/sHLA-B*57:01 and parental *LCL721.221* cells demonstrate the differential regulatory impact of ABC on target cells that are solely distinguished by the expression of the ABC risk allele HLA-B*57:01. The HLA-B*57:01-associated upregulation of distinct proteins might result in a redistribution of the available immunopeptidome and favor the recruitment of neo-antigens by HLA-B*57:01.

IFIH1 encoding the melanoma differentiation-associated protein 5 (MDA5), is significantly upregulated in LCL721.221/sHLA-B*57:01 cells compared to *LCL721.221* cells. MDA5 belongs to the PPR family and is involved in antiviral responses through sensing viral double-stranded RNA (dsDNA) resulting in type I interferon (IFN) production and secretion. MDA5 also plays an essential role in autoimmune diseases, i.e., in multiple sclerosis, psoriasis, rheumatoid arthritis, systemic lupus erythematosus and type 1 diabetes (T1D) [42,43,44,45,46]. The molecular mechanism of how MDA5 contributes to these autoimmune diseases is not fully revealed yet, however it has been proposed that the chronic induction of IFN induces or enhances autoinflammation. SNPs in the *IFIH1* gene resulting in the expression of a truncated, non-functional protein or impaired splicing of the *IFIH1* transcript have been found to be associated with a decreased risk to develop T1D [47]. Conversely, SNPs resulting in increased *IFIH1* transcription and MDA5 expressing or a constitutively active MDA5 protein might lead to autoimmune responses towards cellular dsRNA [48,49,50]. We observed an upregulation of MDA5 in *LCL721.221* cells expressing the ABC risk allele HLA-B*57:01 when compared to parental *LCL721.221* cells following ABC treatment that might favor autoimmune responses triggered by dsRNA derived from cellular sources. Though MDA5 is upregulated in ABC-treated LCL721.221/sHLA-B*57:01, but not in ABC-treated parental *LCL721.221* cells, MDA5 function in autoimmunity seems to be associated with the ABC risk allele HLA-B*57:01.

The PPI network of significantly regulated proteins in LCL721.221/sHLA-B*57:01 cells compared to parental *LCL721.221* cells revealed the involvement of upregulated proteins, particularly in IFN production, RNA and mRNA processing and purine metabolism. In contrast, downregulated proteins are rather involved regulatory processes and small molecule metabolism. IFN production plays a crucial role in the development of systemic autoimmune diseases by activating antigen presenting cells and immune effector cells with potential autoreactive activity [51]. Upregulated proteins in LCL721.221/sHLA-B*57:01 cells compared to parental cells that function in RNA and mRNA processing particularly include proteins belonging to the spliceosome (PRP31, CTNNBL1, SMU1, SF3B6, SRSF2). Spliceosome components play a role in immunity, i.e., the serine/arginine-rich splicing factor 2 (SRSF2) can induce inflammatory cytokine production through TLR-mediated NFкB activation [52].

*IPA* enabled the identification of upstream regulators that might be activated or inhibited by ABC in LCL721.221/sHLA-B*57:01 cells compared to parental cells. CSF1, KAT2 and IRF3 have been identified as activated upstream regulators of various proteins. These proteins play an essential role in innate and adaptive immune responses as well as in inflammatory processes. The cytokine CSF1 (also named macrophage colony-stimulating factor) is a ligand for the tyrosine kinase macrophage-colony stimulating factor 1 receptor (CSF1R) on mononuclear phagocytes and elicits production and differentiation of macrophages [53,54]. Furthermore, CFS1 induces the production of proinflammatory cytokines and chemokines in human whole blood [55] and an involvement in various inflammatory diseases has been described for CSF1 [53]. IRF3 has an essential function in innate antiviral immune responses. Upon recognition of microbial molecular components by PPRs, IRF3 activation leads to the expression of type I IFNs. Overexpression of IRF3 provokes the induction of an efficient antiviral state [56]. Despite the effective function in antiviral immunity, certain IFN-driven autoimmune diseases are caused by unregulated IRF3 [57,58].

Here, we demonstrated the regulatory impact of ABC/HLA-B*57:01. Altogether, we revealed widespread effects of ABC on proteins involved in immune functions and inflammation. This study exemplifies the complexity of regulatory cellular processes induced by drugs that contribute to HLA-mediated ADRs and emphasizes the requirement to fundamentally comprehend molecular and cellular mechanisms affected by drug application to improve drug safety and facilitate personalized medicine.

## Figures and Tables

**Table 1:** Multiple reaction monitoring (MRM) transitions monitored (*m*/*z*) with cone and collision energy.

Analyte	MRM [*m*/*z*]	Dwell [s]	Cone [V]	Collision [eV]
ABC	287.2 → 191.1	0.05	36	20
	287.2 → 78.9	0.05	36	31
ABC-D4	291.3 → 195.1	0.05	37	29
	191.3 → 78.9	0.05	37	20

**Table 2:** Strongest upregulated proteins in LCL721.221/sHLA-B*57:01 cells compared to parental *LCL721.221* cells after ABC treatment.

Protein Name	Gene Code	Regulation	*p*-Value
Histone H3.3	H3F3A	25.63	<0.001
Nucleoside diphosphate kinase A	NME1	8.69	0.003
Interferon-induced helicase C domain-containing protein 1	IFIH1	4.75	0.002
Periphilin-1	PPHLN1	3.92	0.001
Glucosylceramidase	GBA	3.72	0.044
Ras-related protein Rab-35	RAB35	3.71	0.008
Mitochondrial fission process protein 1	MTFP1	3.61	0.045
Hermansky-Pudlak syndrome 5 protein	HPS5	3.14	0.021
Transcription factor A, mitochondrial	TFAM	3.12	0.023
Prefoldin subunit 3	VBP1	2.95	0.010

**Table 3:** Upstream regulator of significantly up- and downregulated proteins in LCL721.221/sHLA-B*57:01 cells compared to parental cells following ABC treatment.

Protein Name of Upstream Regulator	Gene Code	Predicted Activation State	Activation Z-Score	Target Molecules
Macrophage colony-stimulating factor 1	CSF1	Activated	3.411	ACSL4, ATP1B1, CD36, CFL1, CPT2, DHCR7, ETFA, FASN, FDFT1, FDPS, HADHB, HSPD1, IDH3A, IDI1, IQGAP1, LCP1, MT-CO2, RPL5, RRM2, RUVBL2, SCP2, SLC30A1, TFAM, UHRF1
Histone acetyltransferase KAT2A	KAT2	Activated	2.236	HSP90AA1, HSPD1, LDHA, PRKDC, SAE1, XPO1
Interferon regulatory factor 3	IRF3	Activated	2.187	AHNAK, ANXA4, CD58, IFI44L, IFIH1, PTMS, STAT1, TMPO
Signal transducer and activator of transcription 6	STAT6	Inhibited	−2.228	CDK6, G6PD, PFKL, PYGL
DNA binding protein Ikaros	IKZF1	Inhibited	−2.000	AHNAK, ANXA1, CDK2, CTSS, FASN, FSCN1, IFI44L, IFIH1, RAB35, STAT5B, SYNGR2

## Supplementary Materials

The following are available online at https://www.mdpi.com/article/10.3390/jpm12010040/s1, Figure S1: Number of dead cells after treatment of LCL721.221 cells with various ABC concentrations for 24 h. The number of dead cells was calculated by 7-AAD staining and the percentage of dead cells is depicted in the diagram for n = 2. Figure S2: Analysis of the specific cytotoxicity of CD8+ cells from an HLA-B*57:01 carrier towards ABC-treated and untreated LCL721.221/HLA-B*57:01 cells. (A) CD8+ T cells from an HLA-B*57:01 carrier were incubated with LCL721.221/HLA-B*57:01 target cells in a 10:1 ratio for 4 h. ABC-preincubated target cells were previously stained with 1 µM CFSE and untreated target cells were stained with 4 µM CFSE to distinguish between the two target cell populations. After 4 h of incubation, target cell viability of untreated and ABC-treated cells was determined by 7-AAD staining to calculate the cytotoxic potential of CD8^+^ cells. (B) ABC-treated and untreated target cells were incubated for 4 h in the absence of CD8^+^ cells. Afterwards, target cell viability was determined by 7-AAD staining to calculate the spontaneous cell death of untreated and ABC-treated cells.
