# Supplementary material for: Unravelling the Proteomics of HLA-B*57:01+ Antigen Presenting Cells during Abacavir Medication
Source: J Pers Med. 2022 Jan 4;12(1):40. doi: 10.3390/jpm12010040 (PMC8781935; doi:10.3390/jpm12010040)
Supplement: Supplementary file 1 [file jpm-12-00040-s001.zip › jpm-1514792-supplementary.pdf]

Supplementary Materials

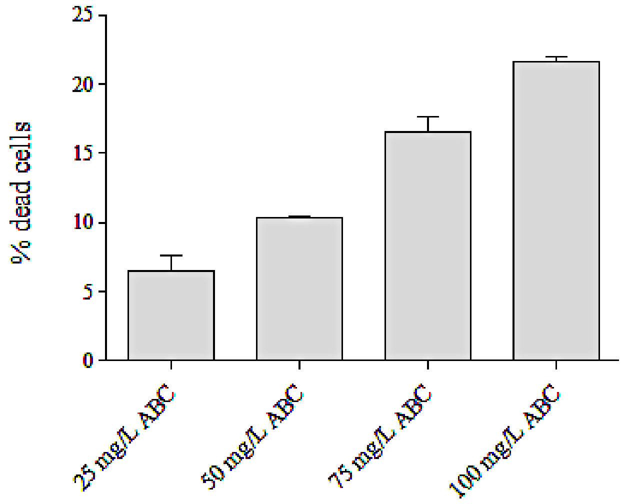

Figure S1: Number of dead cells after treatment of LCL721.221 cells with various ABC concentrations for 24 h. The number of dead cells was calculated by 7-AAD staining and the percentage of dead cells is depicted in the diagram for n=2.

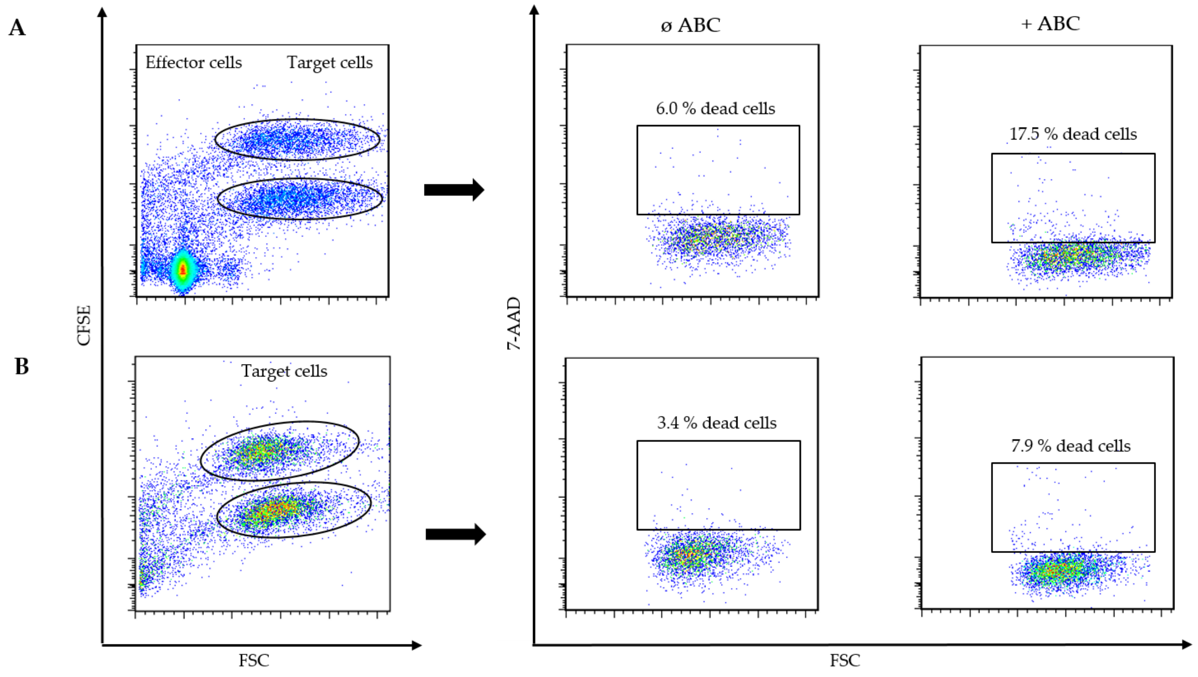

Figure S2: Analysis of the specific cytotoxicity of CD8⁺ cells from an HLA-B*57:01 carrier towards ABC-treated and untreated *LCL721.221/HLA-B*57:01* cells. (**A**) CD8⁺ T cells from an HLA-B*57:01 carrier were incubated with *LCL721.221/HLA-B*57:01* target cells in a 10:1 ratio for 4 hours. ABC-preincubated target cells were previously stained with 1 µM CFSE and untreated target cells were stained with 4 µM CFSE to distinguish between the two target cell populations. After 4 hours of incubation, target cell viability of untreated and ABC-treated cells was determined by 7-AAD staining to calculate the cytotoxic potential of CD8⁺ cells. (**B**) ABC-treated and untreated target cells were incubated for 4 hours in the absence of CD8⁺ cells. Afterwards, target cell viability was determined by 7-AAD staining to calculate the spontaneous cell death of untreated and ABC-treated cells.
